# Advancing Social Protection and Tuberculosis Elimination in India – Beyond Cash Transfers and Towards Addressing Social and Structural Determinants for a Healthier Future; A Response to the Recent Commentaries

**DOI:** 10.34172/ijhpm.2023.8130

**Published:** 2023-06-13

**Authors:** Jigna D. Dave, Mihir P. Rupani

**Affiliations:** ^1^Department of Respiratory Medicine, Government Medical College Bhavnagar, Maharaja Krishnakumarsinhji Bhavnagar University, Bhavnagar, India; ^2^Department of Community Medicine, Government Medical College Bhavnagar, Maharaja Krishnakumarsinhji Bhavnagar University, Bhavnagar, India; ^3^Department of Clinical Epidemiology, Division of Health Sciences, ICMR-National Institute of Occupational Health (NIOH), Indian Council of Medical Research (ICMR), Ahmedabad, India

 In 2022, India witnessed a staggering surge with a record number of 2.42 million reported cases of tuberculosis (TB).^1^ According to recent estimates, the median total costs due to TB among patients in India’s public and private sectors are Indian rupees (INR) 7500 (~US$ 104) and 20 000 (~US$ 277), respectively.^1,2^ The escalating burden of TB in India necessitates comprehensive and cost-effective socioeconomic support measures. Under the direct benefit transfer (DBT) scheme in India, patients with TB are provided a monetary assistance of INR 500 (~US$ 7) which is directly credited to their bank accounts to support their nutritional supplementation needs.^3^ We extend our gratitude to the commentators for their valuable insights on our publication titled “Does direct benefit transfer improve outcomes among people with tuberculosis? – A mixed-methods study on the need for a review of the cash transfer policy in India.”^4-8^ Our study revealed that not all patients received the DBT, and there were delays in its disbursement for those who did receive it.^8^ However, despite these challenges, we observed a positive impact on TB treatment outcomes associated with the receipt of DBT.^8^

 We concur with Ahmad Fuady on the importance of a global agenda and political will to establish an efficient approach for delivering socioeconomic support and nutritional kits to TB-affected households.^4,9,10^ It is imperative to align the cash transfers with the recently determined TB-related patient costs in India.^1,2^ Considering the range of TB costs reported to be between US$ 104-277 in India,^1,2^ the DBT amount of US$ 7 falls significantly short in covering essential expenses, including nutritional supplementation, wage loss, and transportation.^4,11^

 We appreciate the recognition by Rubinstein and Blumenfeld regarding the challenges associated with conducting randomized trials in this context.^5^ They rightly emphasize the significance of observational studies, like ours, and modeling studies that suggest an association between social interventions and treatment outcomes.^5^ We agree with their proposition to promote and expand conditional cash transfer schemes to all TB patients, particularly those in economically vulnerable groups.^12^ Addressing social determinants of health, such as poverty, education, housing, and employment opportunities, is crucial, and we concur with the need to focus on cash transfers as part of the solution.^5^

 Schraufnagel and Shete commend our efforts in evaluating the world’s largest cash transfer program.^6^ They highlight the conflicting evidence in our study, noting that non-receipt of DBT was associated with unfavorable treatment outcomes, while late receipt of first and last instalments showed no association.^6^ They raise questions regarding the mechanism through which DBT affects TB treatment outcomes.^6^ We appreciate their use of the CFIR-ERIC (Consolidated Framework for Implementation Research–Expert Recommendations for Implementing Change) implementation framework to delve into our qualitative data and identify potential mechanisms for the improvement of treatment outcomes.^6^ Using the implementation framework, the commentators have rightly pointed out the possible mechanisms through which DBT might be improving treatment outcomes.^6^ Their suggestions to evaluate DBT in the context of nutritional outcomes, socioeconomic outcomes, satisfaction, and equity are well-taken and would contribute to a better understanding of the implementation science behind DBT.^6^

 Shah brings attention to several implementation challenges of DBT, including registration difficulties due to lack of bank accounts or documents, delays in receipt of DBT, and potential misuse of DBT funds.^7^ In addition, Shah has emphasized the necessity of adjusting the DBT amount to account for inflation since its launch in 2018.^7^ Furthermore, Shah highlights the need to address stigma and proposes overarching activities to tackle social and structural determinants of health with strong political leadership.^7^ We concur with Shah’s suggestions to estimate and project the budget requirements for social support schemes like DBT, improve the health system, provide training for staff involved in DBT disbursements, and establish a target-based evaluation system for the program.^7^

 To enhance the effectiveness of the DBT program for TB patients in India, we emphasize the need to increase the cash assistance amount. Additionally, a separate provision should be made for the direct supply of nutrition kits on a monthly basis, even if in the form of raw items, which patients can redeem at pre-identified local provision stores using vouchers. Ensuring the inclusion of patients undergoing treatment in the private sector within the DBT program is of utmost importance to achieve comprehensive coverage. Strategies such as private practitioners informing the district TB officer and TB health workers collecting relevant information can facilitate the registration of privately treated patients. To address the challenges faced by migrant workers or those moving to other states, providing a card with contact details of the patient as well as the TB health worker would aid in tracking and ensuring continuity of the DBT benefits. Finally, making the DBT program conditional on monthly clinical follow-up at the health facility can help improve treatment adherence.

 It would be intriguing to assess the impact of the scheme launched in September 2022, wherein donors, referred to as ‘Ni-kshay Mitras,’ which include co-operative societies, corporates, elected representatives, individuals, institutions, non-governmental organizations, and political parties, ‘adopt’ patients undergoing TB treatment to provide additional diagnostic, nutritional, and vocational support.^13,14^ Such an evaluation would shed light on the potential effects of this innovative approach on nutritional, socioeconomic, and TB treatment outcomes.^6,13,14^

 In conclusion, we appreciate the valuable insights provided by the commentators and their acknowledgment of the limitations of our study. By carefully considering their suggestions and implementing the proposed improvements, we can enhance the impact of the DBT program and provide better social protection for TB patients in India. It is imperative to address the challenges of cash transfers while also addressing the social determinants of health. With focused efforts and continuous evaluation, we can pave the way forward towards achieving TB elimination in India.

## Ethical issues

 Not applicable.

## Competing interests

 Authors declare that they have no competing interests.
